# Guiding Value of Circulating Tumor Cells for Preoperative Transcatheter Arterial Embolization in Solitary Large Hepatocellular Carcinoma: A Single-Center Retrospective Clinical Study

**DOI:** 10.3389/fonc.2022.839597

**Published:** 2022-05-18

**Authors:** Qiao Zhang, Feng Xia, Ali Mo, Weiming He, Jiazhen Chen, Weiqiao Zhang, Weiqiang Chen

**Affiliations:** ^1^ Zhongshan People’s Hospital, Guangdong Medical University, Zhongshan, China; ^2^ Department of Hepatic Surgery Center, Tongji Hospital of Tongji Medical College of Huazhong University of Science and Technology, Wuhan, China

**Keywords:** preoperative transcatheter arterial embolization, circulating tumor cells, hepatocellular carcinoma, prognosis, TACE

## Abstract

**Background:**

Large hepatocellular carcinoma (LHCC) is highly malignant and prone to recurrence, leading to a poor long-term prognosis for patients. There is an urgent need for measures to intervene in postoperative recurrence. Preoperative Transcatheter Arterial Embolization (TACE) is an effective treatment. However, there is a lack of reliable preoperative indicators to guide the application of preoperative TACE. We, therefore, investigated whether the preoperative status of circulating tumor cells (CTCs) could be used to guide preoperative TACE for HCC treatment.

**Methods:**

This study recruited 361 HCC patients and compared recurrence-free survival (RFS) and overall survival (OS) in patients treated with TACE prior to surgery and those not treated with TACE. Patients were divided into CTC-positive group and CTC-negative group according to CTC status, and the effect of preoperative TACE on RFS and OS was compared in each subgroup.

**Results:**

In CTC-positive patients, preoperative TACE reduces early recurrence and improves long-term survival. However, HCC patients did not benefit from preoperative TACE for the overall population and CTC-negative patients.

**Conclusions:**

Preoperative CTC testing is a reliable indicator of whether HCC patients received TACE preoperatively. CTC positivity was associated with early tumor recurrence, and preoperative TACE could reduce early recurrence and long-term prognosis in CTC-positive patients.

## Introduction

Hepatocellular carcinoma (HCC) is the sixth most common malignancy globally and the third leading cause of cancer deaths (1). For early-stage HCC, partial hepatectomy prolongs disease-free survival (RFS) and overall survival (OS) in HCC patients (2, 3). However, for HCC patients with large hepatocellular carcinoma (> 5 cm), the tumor is highly malignant and prone to recurrence after surgery, with the vast majority of patients eventually dying due to tumor recurrence (4). This creates an urgent need for appropriate treatment measures to control postoperative recurrence of HCC (5).

Previous studies have reported that preoperative transcatheter arterial embolization (TACE) plays an essential role in improving RFS and OS in patients with HCC (6–15). It has also been suggested that preoperative TACE does not improve RFS and OS in HCC patients (16–25). Therefore, many scholars have speculated that preoperative TACE may only benefit certain particular types of HCC groups, especially those with a higher degree of malignancy (26). However, there is no relevant definition of this special type; there is a lack of relevant and reliable preoperative information to distinguish which HCC patients may benefit from preoperative TACE.

A new study from Zhongshan Hospital reports that positive pre-surgical circulating tumor cells (CTCs) are associated with early postoperative recurrence (27). In addition, it can guide whether postoperative adjuvant TACE should be performed to avoid unnecessary postoperative TACE and achieve precise treatment (28). In the same study, we also noted that in the HCC population with positive preoperative CTC testing, postoperative TACE reduced early postoperative recurrence and facilitated the survival of HCC patients (28). We, therefore, envisaged whether preoperative CTC could guide preoperative adjuvant TACE in HCC patients? Our research team carried out a correlation retrospective study based on this.

## Materials and Methods

### Patient Population

This study recruited HCC patients who underwent preoperative CTC testing at the Department of General Surgery I of Zhongshan People’s Hospital from January 2010 to December 2017, with the following inclusion criteria: (1) patients diagnosed with HCC by postoperative pathology, (2) no treatment other than TACE, (3) radical tumor resection (R0) that is, negative macroscopic and microscopic tumor resection margins, (4) complete serological and imaging data, (5) tumor diameter not less than 5 cm, and (6) single tumor. Exclusion criteria were (1) patients younger than 18 years of age, (2) presence of vascular tumor thrombus and distant metastasis, (3) patients with Child-Pugh grade C liver function, (4) no severe vital organ dysfunction, (5) patients who underwent palliative resection, and (6) loss of postoperative follow-up data. The Ethics Committee of Zhongshan People’s Hospital has approved the retrospective study, all patients have signed an informed consent form.

### Data Collection

All patients underwent abdominal enhanced CT or MRI, chest CT or X-ray scan. Laboratory tests include blood routine, liver and kidney function, coagulation function, hepatitis B surface antigen, hepatitis C antibody, AFP, and other examinations. The basic data of the included study population, such as gender, age, hepatitis B surface antigen, hepatitis C antibody, AFP level, alanine aminotransferase (ALT), aspartate aminotransferase (AST), γ-glutamyl aminotransferase (GGT), alkaline phosphatase (ALP), creatinine (Cr), albumin (Alb), total bilirubin (TIBL), direct bilirubin (DIBL), international normalized ratio (INR), platelet count, presence of cirrhosis, Child-Pugh grade, presence of microvascular infiltration (MVI), maximum tumor diameter, pathological classification, extent of liver resection, type of liver resection and other data were collected. Minor liver resection was defined as resection of fewer than three Couinaud liver segments, while major liver resection was defined as resection of three or more liver segments. Non-anatomical liver resection included a limited resection or wedge resection; anatomical resections were defined by the Brisbane 2000 system. The continuous variables are transformed into binary variables, and the cut-off value is the upper and lower lines of the recognized normal value.

### Preoperative TACE

Considering that this was a retrospective study, the decision to use TACE prior to surgery was left to the discretion of the treating surgeon and the patient at that time. The patient was placed supine, locally disinfected, draped, and given local anesthetized. The puncture site was chosen to be 2 cm below the inguinal ligament, and the catheter sheath was placed into the femoral artery using the Seldinger technique. Firstly, the DSA technique helps with abdominal trunk and standard hepatic artery angiography to determine the tumor’s location, size, and condition of the tumor. Once the tumor is understood, the catheter sheath is continued deeper into the left or right hepatic artery or the vessel that feeds the tumor, 5-fluorouracil (500 mg/m2) or oxaliplatin (100 mg/m2) was injected into the proper hepatic artery, and embolization was performed using different embolization materials. Patients were asked to return to the hospital 4-6 weeks after embolization for follow-up serology, including blood routine, liver and kidney function, coagulation function, AFP, and imaging included abdominal enhanced CT or MRI, chest X-ray scan, etc. All of the above procedures were performed by highly qualified attending physicians who received relevant interventional medicine.

### Isolation and Identification of CTC

The Cyttel method is used to detect CTCs, and its main principles include the negative immunomagnetic particle assay and immunofluorescence *in situ* hybridization (im-FISH). Jiangsu Lyle Biomedical Technology Co manufactures the kit. For patients with preoperative TACE, samples were obtained within three days before TACE, while for patients without preoperative TACE, the sample extraction must also be completed within three days before surgery. Generally, we draw 5ml peripheral blood, and process the samples strictly according to the manufacturer’s instructions. Firstly, the samples was treated with negative immunomagnetic powder method to remove leukocytes from the peripheral blood, and isolate rare cells in the blood, and finally obtain CTCs. Then, the im-FISH technique was used to fix and dehydrate the samples, then hybridization with chromosome centromeres 1 and 8, followed by sealing with 4-diamidine-2-phenylindole (DAPI) staining solution, and then observation and counting under a fluorescence microscope (29–32). It defined CTC count ≥1 as CTC-positive (32).

### Follow-Up

Each follow-up visit for all patients include AFP, routine blood tests, liver and kidney function tests, coagulation function tests. Enhanced CT or MRI of the abdomen, chest CT, and the bone scan will be performed if tumor residue and signs of tumor recurrence are suspected. The first postoperative follow-up visit would performed one month after the operation. The follow-up frequency was once every 2-3 months within six months after the operation, once every 3-4 months within 6-24 months after the operation, and once every 4-6 months after 24 months after the operation. After a recurrence of HCC, treatment options are chosen according to the recurrence and the patient’s general condition. Treatment options include surgical re-resection, radiofrequency ablation (RFA), percutaneous ethanol injection (PEI), TACE, taking targeted drugs, immune drug therapy, and even liver transplantation. OS was defined as the date of surgery until patient death or last follow-up, and RFS was defined as the date of surgery until patient signs of recurrence or last follow-up. Recurrence was classified as early recurrence and late recurrence using a cut-off value of 24 months.

### Statistical Analysis

Continuous variables were expressed as median ± square difference (Median ± SD), and categorical variables were expressed as number (n) or percentage (%) of patients. The t-test or Mann-Whitney test was used to compare two groups of continuous variables, and the χ2 or Fisher’s exact test was used to compare two groups of categorical variables. The survival curves of OS and RFS of the patients were plotted using the Kaplan-Meier method, and the OS and RFS of the patients in the preoperative TACE group and the two groups without preoperative TACE were compared using the log-rank. We also used the Landmark analysis method to analyze the results of assessing early recurrence (recurrence 24 months after surgery) and late recurrence. Univariate and multivariate Cox regression models were used to analyze the independent risk factors of each factor on patients’ RFS and OS. All statistics and graphs for this study were completed in R (version 3.62). P values < 0.05 were considered statistically significant.

## Results

### Characteristics of Patients With HCC

**Figure 1:**
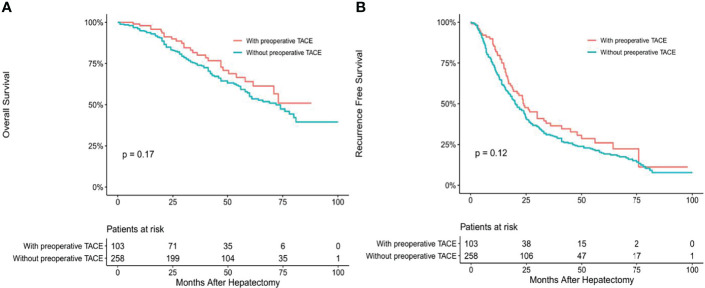
Overall **(A)** and recurrence-free **(B)** survival curves of overall HCC patients with or without preoperative TACE.

Baseline characteristics of the total population of HCC are listed in **Table 1**. The population was divided into positive and negative subgroups based on the preoperative CTCs count. The clinical baseline of each subgroup is shown in **Table 2**. In this study, a total of 361 patients with HCC were enrolled in this study, including 211 patients of CTC-positive (58.4%) and 103 patients of preoperative TACE (28.5%). The median follow-up time of the CTC-positive group was 38.0 months, while the median follow-up time of the CTC-negative group was 44.5 months. The median follow-up time of HCC patients with preoperative TACE was 41.0 months, while patients without TACE were 36.5 months. During follow-up, 134 patients died, and 275 patients developed tumor recurrence. In the CTC-positive and CTC-negative subgroups, the clinicopathological variables were similar, comparable and not statistically significant between patients who underwent preoperative TACE and those who did not (*P* > 0.05; **Table 2**). In the overall population, RFS and OS were similar of patients with and without preoperative TACE; Preoperative TACE did not improve the prognosis of HCC (*P* > 0.05; **Figures 1A, B**).

**Table 1:** Baseline characteristics of HCC patients for the overall population.

Variable		n = 361	
		n	%
**Age (years)**	<60	217	60.1
	≥60	144	39.9
**Gender**	Female	83	23.0
	Male	278	77.0
**CTC**	Negative	150	41.6
	Positive	211	58.4
**HBV**	No	16	4.4
	Yes	345	95.6
**HCV**	No	355	98.3
	Yes	6	1.7
**Cirrhosis**	No	115	31.9
	Yes	246	68.1
**Child-Pugh**	A	313	86.7
	B	48	13.3
**ALT (U/L)**	<50	231	64.0
	≥50	130	36.0
**AST (U/L)**	<40	126	34.9
	≥40	235	65.1
**GGT (U/L)**	<45	77	21.3
	≥45	284	78.7
**ALP (U/L)**	<125	276	76.5
	≥125	85	23.5
**Alb (g/L)**	<35	55	15.2
	≥35	306	84.8
**TIBL (umol/L)**	<20.4	300	83.1
	≥20.4	61	16.9
**DIBL (umol/L)**	<6.8	289	80.1
	≥6.8	72	19.9
**CR (umol/L**	<84	305	84.5
	≥84	56	15.5
**INR**	<1.15	248	68.7
	≥1.15	113	31.3
**PLT (10^9^/L)**	<100	100	27.7
	≥100	261	72.3
**AFP (ug/mL)**	<400	141	39.1
	≥400	220	60.9
**Tumor diameter (cm)**	<10	207	57.3
	≥10	154	42.7
**Edmondson stage**	I+II	57	15.8
	III+IV	304	84.2
**MVI**	No	141	39.1
	Yes	220	60.9
**Tumor capsule**	Complete	84	23.3
	Absent or Partial	277	76.7
**Extent of liver resection**	Major liver resection	232	64.3
	Minor liver resection	129	35.7
**Type of liver resection**	Anatomical	139	38.5
	Non-anatomical	222	61.5
**Postoperative TACE**	No	181	50.5
	Yes	180	49.5
**Site of recurrence**	Intrahepatic	217	78.9
	Extrahepatic	28	10.2
	Intrahepatic and extrahepatic	30	10.9
**Preoperative TACE**	No	258	71.5
	Yes	103	28.5

AST, aspartate aminotransferase; ALT, alanine aminotransferase; GGT, gamma glutamyl transpeptidase; ALP, alkaline phosphatase; Alb, albumin; TIBL, total bilirubin; DIBL, direct bilirubin; CR, creatinine; INR, international normalized ratio; PLT, blood platelet; AFP, alpha fetoprotein; HBV, hepatitis B virus; HCV, hepatitis C virus; CTC, circulating tumor cells; MVI, microvascular invasion.

**Table 2:** Comparison of clinicopathological variables between preoperative TACE and control group in HCC patients with CTC-positive and CTC-negative groups.

Variable		CTC Positive (n=211)			CTC Negative (n=150)		
		Non-TACE (n=148)	TACE (n=63)	*p*	Non-TACE (n=110)	TACE (n=40)	*p*
**Age (years)**	<60	88 (59.5)	39 (61.9)	0.858	63 (57.3)	27 (67.5)	0.346
	≥60	60 (40.5)	24 (38.1)		47 (42.7)	13 (32.5)	
**Gender**	Female	41 (27.7)	13 (20.6)	0.366	25 (22.7)	4 (10.0)	0.131
	Male	107 (72.3)	50 (79.4)		85 (77.3)	36 (90.0)	
**HBV**	No	10 (6.8)	1 (1.6)	0.227	5 (4.5)	0 (0.0)	0.391
	Yes	138 (93.2)	62 (98.4)		105 (95.5)	40 (100.0)	
**HCV**	No	144 (97.3)	62 (98.4)	1.000	109 (99.1)	40 (100.0)	1.000
	Yes	4 (2.7)	1 (1.6)		1 (0.9)	0 (0.0)	
**Cirrhosis**	No	49 (33.1)	18 (28.6)	0.627	36 (32.7)	12 (30.0)	0.905
	Yes	99 (66.9)	45 (71.4)		74 (67.3)	28 (70.0)	
**Child-Pugh**	A	129 (87.2)	52 (82.5)	0.506	98 (89.1)	34 (85.0)	0.691
	B	19 (12.8)	11 (17.5)		12 (10.9)	6 (15.0)	
**ALT (U/L)**	<50	90 (60.8)	40 (63.5)	0.832	77 (70.0)	24 (60.0)	0.338
	≥50	58 (39.2)	23 (36.5)		33 (30.0)	16 (40.0)	
**AST (U/L)**	<40	48 (32.4)	22 (34.9)	0.848	42 (38.2)	14 (35.0)	0.869
	≥40	100 (67.6)	41 (65.1)		68 (61.8)	26 (65.0)	
**GGT (U/L)**	<45	32 (21.6)	16 (25.4)	0.675	22 (20.0)	7 (17.5)	0.913
	≥45	116 (78.4)	47 (74.6)		88 (80.0)	33 (82.5)	
**ALP (U/L)**	<125	113 (76.4)	48 (76.2)	1.000	86 (78.2)	29 (72.5)	0.611
	≥125	35 (23.6)	15 (23.8)		24 (21.8)	11 (27.5)	
**Alb (g/L)**	<35	25 (16.9)	13 (20.6)	0.651	12 (10.9)	5 (12.5)	1.000
	≥35	123 (83.1)	50 (79.4)		98 (89.1)	35 (87.5)	
**TIBL (umol/L)**	<20.4	123 (83.1)	53 (84.1)	1.000	92 (83.6)	32 (80.0)	0.782
	≥20.4	25 (16.9)	10 (15.9)		18 (16.4)	8 (20.0)	
**DIBL (umol/L)**	<6.8	118 (79.7)	49 (77.8)	0.893	91 (82.7)	31 (77.5)	0.624
	≥6.8	30 (20.3)	14 (22.2)		19 (17.3)	9 (22.5)	
**CR (umol/L)**	<84	130 (87.8)	51 (81.0)	0.273	92 (83.6)	32 (80.0)	0.782
	≥84	18 (12.2)	12 (19.0)		18 (16.4)	8 (20.0)	
**INR**	<1.15	99 (66.9)	47 (74.6)	0.343	79 (71.8)	23 (57.5)	0.143
	≥1.15	49 (33.1)	16 (25.4)		31 (28.2)	17 (42.5)	
**PLT (10^9^/L)**	<100	44 (29.7)	16 (25.4)	0.637	25 (22.7)	15 (37.5)	0.109
	≥100	104 (70.3)	47 (74.6)		85 (77.3)	25 (62.5)	
**AFP (ug/mL)**	<400	54 (36.5)	31 (49.2)	0.116	43 (39.1)	13 (32.5)	0.584
	≥400	94 (63.5)	32 (50.8)		67 (60.9)	27 (67.5)	
**Tumor diameter (cm)**	<10	69 (46.6)	33 (52.4)	0.538	74 (67.3)	24 (60.0)	0.526
	≥10	79 (53.4)	30 (47.6)		36 (32.7)	16 (40.0)	
**Edmondson stage**	I+II	20 (13.5)	8 (12.7)	1.000	24 (21.8)	5 (12.5)	0.296
	III+IV	128 (86.5)	55 (87.3)		86 (78.2)	35 (87.5)	
**MVI**	No	53 (35.8)	19 (30.2)	0.526	48 (43.6)	21 (52.5)	0.437
	Yes	95 (64.2)	44 (69.8)		62 (56.4)	19 (47.5)	
**Tumor capsule**	Complete	121 (81.8)	45 (71.4)	0.136	31 (28.2)	8 (20.0)	0.424
	Absent or Partial	27 (18.2)	18 (28.6)		79 (71.8)	32 (80.0)	
**Extent of liver resection**	Major liver resection	94 (63.5)	44 (69.8)	0.468	68 (61.8)	26 (65.0)	0.869
	Minor liver resection	54 (36.5)	19 (30.2)		42 (38.2)	14 (35.0)	
**Type of liver resection**	Anatomical	57 (38.5)	23 (36.5)	0.905	42 (38.2)	17 (42.5)	0.772
	Non-anatomical	91 (61.5)	40 (63.5)		68 (61.8)	23 (57.5)	
**Postoperative TACE**	No	72 (48.6)	33 (52.4)	0.730	58 (52.7)	18 (45.0)	0.514
	Yes	76 (51.4)	30 (47.6)		52 (47.3)	22 (55.0)	
**Site of recurrence**	Intrahepatic	111 (82.2)	26 (72.2)	0.288	62 (78.5)	18 (72.0)	0.599
	Extrahepatic	11 (8.1)	6 (16.7)		7 (8.9)	4 (16.0)	
	Intrahepatic and extrahepatic	13 (9.6)	4 (11.1)		10 (12.7)	3 (12.0)	

TACE, transcatheter arterial chemoembolization; AST, aspartate aminotransferase; ALT, alanine aminotransferase; GGT, gamma glutamyl transpeptidase; ALP, alkaline phosphatase; AlB, albumin; TIBL, total bilirubin; DIBL, direct bilirubin; CR, creatinine; INR, international normalized ratio; PLT, blood platelet; AFP, alpha fetoprotein; HBV, hepatitis B virus; HCV, hepatitis C virus; HCC, hepatocellular carcinoma; CTC, circulating tumor cells; MVI, microvascular invasion.

### CTCs Status Affects OS and RFS of HCC Patients

Using survival curves drawn by the Kaplan Meier method, we found that OS (median 39 months vs. 47 months, *P* < 0.05, **Supplementary Figure 1A**) and RFS (median 17.0 months vs. 24 months, *P* < 0.05 **Supplementary Figure 1B**) in CTC-positive group were worse than those in CTC-negative group. We also analyzed the effect of CTCs status on postoperative recurrence patterns using the landmark method. Using a 24-month cut-off, postoperative recurrence was divided into an early recurrence and late recurrence. We found that CTC positive was associated with postoperative early recurrence (*P* < 0.05; **Supplementary Figure 2**) but not with late recurrence (*P* > 0.05; **Supplementary Figure 2**).

### The Clinical Efficacy of Preoperative TACE Was Evaluated in Subgroups of CTC-Positive and CTC-Negative Groups

**Figure 2:**
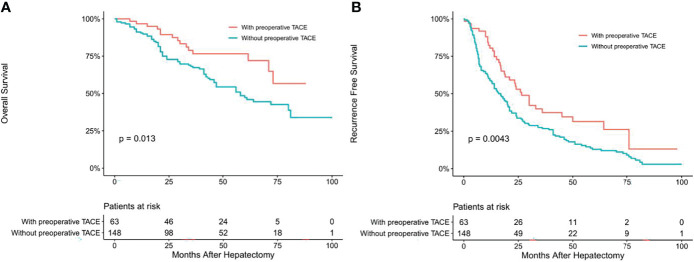
Overall **(A)** and recurrence-free **(B)** survival curves of HCC patients in CTC-positive patients with or without preoperative TACE.

**Figure 3:**
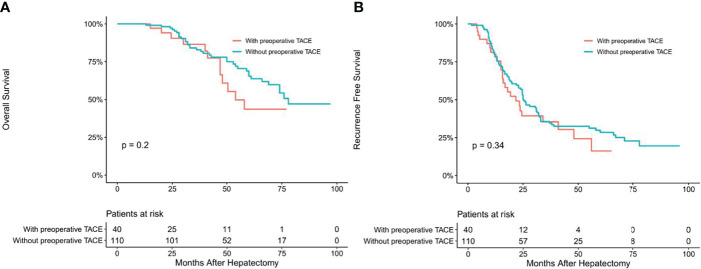
Overall **(A)** and recurrence-free **(B)** survival curves of HCC patients in CTC-negative patients with or without preoperative TACE.

To determine whether CTCs status affects the clinical efficacy of TACE, we stratified patients’ CTCs status of and compared the OS and RFS between patients with and without preoperative TACE at different CTCs status. In the CTC-positive group, preoperative TACE prolonged OS and RFS in HCC patients; the difference was statistically significant (*P* < 0.05; **Figures 2A, B**). In CTC-negative group, preoperative TACE could not improve RFS and OS, and the difference was not statistically significant (*P* > 0.05; **Figures 3A, B**).

Univariate and multivariate Cox regression analysis also showed that in CTC-positive group, non-preoperative TACE was an independent risk factor for OS (hazard ratio [HR]= 2.330, 95% confidence interval [*CI*], 1.318-4.120, *P* <0.05; **Table 3**) and RFS(hazard ratio [HR]= 2.332, 95% confidence interval [CI], 1.584-3.432, *P* <0.05; **Table 4**) of HCC patients, while in CTC-negative group, preoperative TACE had no effect on OS (hazard ratio [HR]= 0.655, 95% confidence interval [CI], 0.338-1.267, *P* >0.05; **Table 5**)and RFS (hazard ratio [HR]= 0.805, 95% confidence interval [CI], 0.511-1.269,*P* >0.05; **Table 6**) of HCC patients.

**Table 3:** Univariate and multivariate Cox regression analyses were used to identify independent risk factors for overall survival in CTC-positive patients.

Variables	HR comparison	UV HR (95% *CI*)	UV p	MV HR (95% *CI*)	MV p*
**Preoperative TACE**	No vs. yes	2.006 (1.149-3.503)	0.014	2.33 (1.318-4.12)	0.004
**Age**	≥60 vs <60 years	1.103 (0.716-1.699)	0.656		
**Gender**	Male vs. female	1.36 (0.808-2.287)	0.247		
**HBV**	Yes vs. no	2.131 (0.671-6.761)	0.199		
**HCV**	Yes vs. no	0 (0-Inf)	0.996		
**Cirrhosis**	Yes vs. no	0.965 (0.605-1.539)	0.881		
**Child Pugh**	B vs A	1.436 (0.778-2.649)	0.247		
**ALT**	≥50 vs <50 U/L	1.138 (0.74-1.748)	0.557		
**AST**	≥40 vs <40 U/L	0.963 (0.615-1.506)	0.867		
**GGT**	≥45 vs <45 U/L	1.435 (0.819-2.513)	0.206		
**ALP**	≥40 vs <40 U/L	0.857 (0.514-1.428)	0.553		
**Alb**	≥35 vs <35 g/L	0.965 (0.552-1.686)	0.900		
**TIBL**	≥20.4 vs <20.4 umol/L	0.9 (0.489-1.656)	0.734		
**DIBL**	≥6.8 vs <6.8 umol/L	1.033 (0.607-1.757)	0.904		
**CR**	≥80.4 vs <80.4 umol/L	0.845 (0.448-1.593)	0.602		
**INR**	≥1.15 vs <1.15	1.01 (0.637-1.602)	0.966		
**PLT**	≥ 100 vs <100 × 10^9^/L	1.269 (0.769-2.093)	0.352		
**AFP**	≥400 vs <400ng/mL	3.137 (1.904-5.168)	<0.001	1.925 (1.129-3.28)	0.016
**Tumor diameter**	<10 vs ≥10cm	0.508 (0.331-0.779)	0.002	0.53 (0.337-0.833)	0.006
**Edmondson stage**	III+IV vs I+II	2.814 (1.345-5.89)	0.006	2.864 (1.348-6.081)	0.006
**MVI**	Yes vs. no	3.311 (1.912-5.736)	<0.001	2.159 (1.193-3.907)	0.011
**Tumor capsule**	Complete vs. incomplete	0.857 (0.514-1.429)	0.553		
**Extent of liver resection**	Major vs. minor	0.877 (0.562-1.367)	0.562		
**Type of liver resection**	Anatomical vs. non-anatomical	0.876 (0.572-1.339)	0.469		
**Postoperative TACE**	Yes vs. no	0.815 (0.533-1.247)	0.346		

TACE, transcatheter arterial chemoembolization; AST, aspartate aminotransferase; ALT, alanine aminotransferase; GGT, gamma glutamyl transpeptidase; ALP, alkaline phosphatase; Alb, albumin; TIBL, total bilirubin; DIBL, direct bilirubin; CR, creatinine; INR, international normalized ratio; PLT, blood platelet; AFP, alpha fetoprotein; HBV, hepatitis B virus; HCV, hepatitis C virus; MVI, microvascular invasion; CI, confidence interval; HR, hazard ratio; UV, univariable; MV, multivariable.

*Those variables found significant at p < 0.05 in univariable analyses were entered into multivariable Cox-regression analyses.

**Table 4:** Univariate and multivariate Cox regression analyses were used to identify independent risk factors for recurrence free survival in CTC positive patients.

Variables	HR comparison	UV HR (95% *CI*)	UV p	MV HR (95% *CI*)	MV p*
Preoperative TACE	No vs. yes	1.699 (1.175-2.455)	0.005	2.332 (1.584-3.432)	≤0.001
Age	≥60 vs <60 years	1.307 (0.953-1.793)	0.097		
Gender	Male vs. female	0.931 (0.662-1.309)	0.680		
HBV	Yes vs. no	0.961 (0.517-1.786)	0.899		
HCV	Yes vs. no	1.533 (0.627-3.746)	0.349		
Cirrhosis	Yes vs. no	0.816 (0.593-1.123)	0.211		
Child Pugh	B vs A	1.426 (0.909-2.237)	0.122		
ALT	≥50 vs <50 U/L	1.171 (0.861-1.594)	0.315		
AST	≥40 vs <40 U/L	1.062 (0.769-1.465)	0.716		
GGT	≥45 vs <45 U/L	1.373 (0.939-2.008)	0.102		
ALP	≥40 vs <40 U/L	1.268 (0.892-1.803)	0.186		
Alb	≥35 vs <35 g/L	0.94 (0.636-1.388)	0.755		
TIBL	≥20.4 vs <20.4 umol/L	0.941 (0.622-1.422)	0.772		
DIBL	≥6.8 vs <6.8 umol/L	1.01 (0.692-1.472)	0.961		
CR	≥80.4 vs <80.4 umol/L	1.185 (0.774-1.814)	0.434		
INR	≥1.15 vs <1.15	1.065 (0.771-1.47)	0.703		
PLT	≥ 100 vs <100 × 10^9^/L	1.253 (0.886-1.772)	0.202		
AFP	≥400 vs <400ng/mL	2.617 (1.88-3.643)	<0.001	1.727 (1.214-2.457)	0.002
**Tumor diameter**	<10 vs ≥10cm	0.468 (0.344-0.636)	<0.001	0.472 (0.34-0.655)	<0.001
Edmondson stage	III+IV vs I+II	1.926 (1.202-3.085)	0.006	1.867 (1.154-3.022)	0.011
MVI	Yes vs. no	2.708 (1.917-3.826)	<0.001	2.03 (1.394-2.958)	<0.001
Tumor capsule	Complete vs. incomplete	0.711 (0.491-1.028)	0.070		
**Extent of liver resection**	Major vs. minor	0.915 (0.666-1.257)	0.584		
**Type of liver resection**	Anatomical vs. non-anatomical	1.124 (0.821-1.538)	0.465		
**Postoperative TACE**	Yes vs. no	1.111 (0.821-1.505)	0.494		

TACE, transcatheter arterial chemoembolization; AST, aspartate aminotransferase; ALT, alanine aminotransferase; GGT, gamma glutamyl transpeptidase; ALP, alkaline phosphatase; Alb, albumin; TIBL, total bilirubin; DIBL, direct bilirubin; CR, creatinine; INR, international normalized ratio; PLT, blood platelet; AFP, alpha fetoprotein; HBV, hepatitis B virus; HCV, hepatitis C virus; MVI, microvascular invasion; CI, confidence interval; HR, hazard ratio; UV, univariable; MV, multivariable.

*Those variables found significant at p < 0.05 in univariable analyses were entered into multivariable Cox-regression analyses.

**Table 5:** Univariate and multivariate Cox regression analyses were used to identify independent risk factors for overall survival in CTC-negative patients.

Variables	HR comparison	UV HR (95% *CI*)	UV p	MV HR (95% *CI*)	MV p*
Preoperative TACE	No vs. yes	0.655 (0.338-1.267)	0.209		
Age	≥60 vs <60 years	1.215 (0.689-2.142)	0.500		
Gender	Male vs. female	1.398 (0.653-2.99)	0.388		
HBV	Yes vs. no	0.709 (0.22-2.287)	0.565		
HCV	Yes vs. no	4.328 (0.586-31.979)	0.151		
Cirrhosis	Yes vs. no	0.896 (0.486-1.652)	0.724		
Child Pugh	B vs A	1.833 (0.767-4.38)	0.173		
ALT	≥50 vs <50 U/L	0.763 (0.413-1.409)	0.387		
AST	≥40 vs <40 U/L	1.674 (0.884-3.169)	0.114		
GGT	≥45 vs <45 U/L	1.359 (0.61-3.031)	0.453		
ALP	≥40 vs <40 U/L	1.109 (0.565-2.175)	0.763		
Alb	≥35 vs <35 g/L	0.786 (0.352-1.755)	0.557		
TIBL	≥20.4 vs <20.4 umol/L	0.68 (0.305-1.518)	0.346		
DIBL	≥6.8 vs <6.8 umol/L	0.825 (0.398-1.708)	0.604		
CR	≥80.4 vs <80.4 umol/L	0.645 (0.274-1.52)	0.316		
INR	≥1.15 vs <1.15	0.76 (0.387-1.491)	0.424		
PLT	≥ 100 vs <100 × 10^9^/L	2.266 (0.962-5.335)	0.061		
AFP	≥400 vs <400ng/mL	2.057 (1.069-3.955)	0.031	2.104 (1.092-4.055)	0.026
**Tumor diameter**	<10 vs ≥10cm	0.388 (0.219-0.69)	0.001	0.524 (0.293-0.937)	0.029
Edmondson stage	III+IV vs I+II	3.463 (1.461-8.209)	0.005	4.035 (1.662-9.8)	0.002
MVI	Yes vs. no	4.072 (2.101-7.894)	<0.001	4.007 (2.026-7.926)	<0.001
Tumor capsule	Complete vs. incomplete	1.024 (0.56-1.873)	0.939		
**Extent of liver resection**	Major vs. minor	1.402 (0.792-2.484)	0.246		
**Type of liver resection**	Anatomical vs. non-anatomical	1.763 (0.933-3.333)	0.081		
**Postoperative TACE**	Yes vs. no	1.431 (0.808-2.533)	0.219		

TACE, transcatheter arterial chemoembolization; AST, aspartate aminotransferase; ALT, alanine aminotransferase; GGT, gamma glutamyl transpeptidase; ALP, alkaline phosphatase; Alb, albumin; TIBL, total bilirubin; DIBL, direct bilirubin; CR, creatinine; INR, international normalized ratio; PLT, blood platelet; AFP, alpha fetoprotein; HBV, hepatitis B virus; HCV, hepatitis C virus; MVI, microvascular invasion; CI, confidence interval; HR, hazard ratio; UV, univariable; MV, multivariable.

*Those variables found significant at p < 0.05 in univariable analyses were entered into multivariable Cox-regression analyses.

**Table 6:** Univariate and multivariate Cox regression analyses were used to identify independent risk factors for recurrence free survival in CTC negative patients.

Variables	HR comparison	UV HR (95% *CI*)	UV p	MV HR (95% *CI*)	MV p*
Preoperative TACE	No vs. yes	0.805 (0.511-1.269)	0.351		
Age	≥60 vs <60 years	1.092 (0.739-1.614)	0.658		
Gender	Male vs. female	1.236 (0.751-2.033)	0.404		
HBV	Yes vs. no	0.465 (0.188-1.151)	0.098		
HCV	Yes vs. no	3.417 (0.469-24.914)	0.225		
Cirrhosis	Yes vs. no	1.17 (0.765-1.788)	0.470		
Child Pugh	B vs A	1.244 (0.679-2.278)	0.479		
ALT	≥50 vs <50 U/L	0.682 (0.445-1.043)	0.078		
AST	≥40 vs <40 U/L	0.912 (0.616-1.35)	0.646		
GGT	≥45 vs <45 U/L	0.772 (0.481-1.237)	0.281		
ALP	≥40 vs <40 U/L	1.288 (0.821-2.023)	0.271		
Alb	≥35 vs <35 g/L	1.251 (0.651-2.403)	0.502		
TIBL	≥20.4 vs <20.4 umol/L	1.035 (0.622-1.721)	0.894		
DIBL	≥6.8 vs <6.8 umol/L	1.066 (0.659-1.723)	0.795		
CR	≥80.4 vs <80.4 umol/L	0.671 (0.387-1.163)	0.155		
INR	≥1.15 vs <1.15	0.948 (0.622-1.444)	0.803		
PLT	≥ 100 vs <100 × 10^9^/L	1.101 (0.706-1.717)	0.671		
AFP	≥400 vs <400ng/mL	4.237 (2.61-6.878)	<0.001	4.291 (2.630-7.000)	<0.001
**Tumor diameter**	<10 vs ≥10cm	0.545 (0.363-0.82)	0.004	0.536 (0.353-0.814)	0.003
Edmondson stage	III+IV vs I+II	1.798 (1.079-2.996)	0.024	1.82 (1.089-3.042)	0.022
MVI	Yes vs. no	2.605 (1.732-3.919)	<0.001	2.211 (1.465-3.337)	<0.001
Tumor capsule	Complete vs. incomplete	0.823 (0.527-1.284)	0.390		
**Extent of liver resection**	Major vs. minor	0.875 (0.588-1.303)	0.512		
**Type of liver resection**	Anatomical vs. non-anatomical	1.335 (0.895-1.992)	0.157		
**Postoperative TACE**	Yes vs. no	1.254 (0.852-1.844)	0.251		

TACE, transcatheter arterial chemoembolization; AST, aspartate aminotransferase; ALT, alanine aminotransferase; GGT, gamma glutamyl transpeptidase; ALP, alkaline phosphatase; Alb, albumin; TIBL, total bilirubin; DIBL, direct bilirubin; CR, creatinine; INR, international normalized ratio; PLT, blood platelet; AFP, alpha fetoprotein; HBV, hepatitis B virus; HCV, hepatitis C virus; MVI, microvascular invasion; CI, confidence interval; HR, hazard ratio; UV, univariable; MV, multivariable.

*Those variables found significant at p < 0.05 in univariable analyses were entered into multivariable Cox-regression analyses.

### Preoperative Adjuvant TACE Can Reduce the Early Recurrence of CTC-Positive Patients

**Figure 4:**
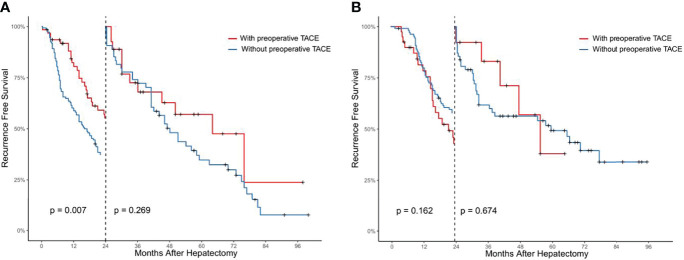
Analysis of the effect of preoperative TACE on early and late postoperative recurrence in CTC positive **(A)** and CTC negative **(B)** HCC patients by landmark method.

Using landmark analysis and taking 24 months as the cutoff value, we found that preoperative TACE could reduce the early recurrence of patients in CTC-positive group (*P* < 0.05, **Figure 4A**), but could not improve the late recurrence rate of patients (*P* > 0.05, **Figure 4A**). In the CTC-negative group, preoperative TACE could not improve the early and late recurrence (*P* > 0.05, **Figure 4B**).

### The Clinicopathological Baseline of CTC-Positive Group and CTC-Negative Group Were Compared

The comparison of clinicopathological variables between the CTC-positive group and CTC-negative group is shown in **Table 7**; the proportion of patients with tumor diameter ≥10cm (48.3.1% vs. 34.7%, *P* < 0.05; **Table 7**) and positive rate of MVI (65.9% vs. 54.0%, *P* < 0.05; **Table 7**) in CTC-positive group was higher than that in CTC-negative group. At the same time, other clinicopathological indicators such as age, sex, HBV, cirrhosis, Child-Pugh, Edmondson stage, and AFP≥400ng/ml were not significantly different(*P* > 0.05; **Table 7**).

**Table 7:** Relationship between positive and negative CTC and clinicopathological variables.

Variable		Overall (361)	CTC- Negative (n=150)	CTC- Positive (n=211)	*p*
Age (%)	<60years	217 (60.1)	90 (60.0)	127 (60.2)	1.000
	≥60years	144 (39.9)	60 (40.0)	84 (39.8)	
Gender (%)	Female	83 (23.0)	29 (19.3)	54 (25.6)	0.206
	Male	278 (77.0)	121 (80.7)	157 (74.4)	
HBV (%)	No	16 (4.4)	5 (3.3)	11 (5.2)	0.551
	Yes	345 (95.6)	145 (96.7)	200 (94.8)	
HCV (%)	No	355 (98.3)	149 (99.3)	206 (97.6)	0.407
	Yes	6 (1.7)	1 (0.7)	5 (2.4)	
Cirrhosis (%)	No	115 (31.9)	48 (32.0)	67 (31.8)	1.000
	Yes	246 (68.1)	102 (68.0)	144 (68.2)	
Child Pugh (%)	A	313 (86.7)	132 (88.0)	181 (85.8)	0.65
	B	48 (13.3)	18 (12.0)	30 (14.2)	
ALT (%)	<50U/L	231 (64.0)	101 (67.3)	130 (61.6)	0.315
	≥50U/L	130 (36.0)	49 (32.7)	81 (38.4)	
AST (%)	<40U/L	126 (34.9)	56 (37.3)	70 (33.2)	0.481
	≥40U/L	235 (65.1)	94 (62.7)	141 (66.8)	
GGT (%)	<45U/L	77 (21.3)	29 (19.3)	48 (22.7)	0.515
	≥45U/L	284 (78.7)	121 (80.7)	163 (77.3)	
ALP (%)	<125U/L	276 (76.5)	115 (76.7)	161 (76.3)	1.000
	≥125U/L	85 (23.5)	35 (23.3)	50 (23.7)	
Alb (%)	<35g/l	55 (15.2)	17 (11.3)	38 (18.0)	0.112
	≥35g/l	306 (84.8)	133 (88.7)	173 (82.0)	
TIBL (%)	<20.4umol/L	300 (83.1)	124 (82.7)	176 (83.4)	0.965
	≥20.4umol/L	61 (16.9)	26 (17.3)	35 (16.6)	
DIBL (%)	<6.8umol/L	289 (80.1)	122 (81.3)	167 (79.1)	0.705
	≥6.8umol/L	72 (19.9)	28 (18.7)	44 (20.9)	
CR (%)	<84umol/L	305 (84.5)	124 (82.7)	181 (85.8)	0.510
	≥84umol/L	56 (15.5)	26 (17.3)	30 (14.2)	
INR (%)	<1.15	248 (68.7)	102 (68.0)	146 (69.2)	0.900
	≥1.15	113 (31.3)	48 (32.0)	65 (30.8)	
PLT (%)	<100	100 (27.7)	40 (26.7)	60 (28.4)	0.802
	≥100	261 (72.3)	110 (73.3)	151 (71.6)	
AFP (%)	<400ug/mL	141 (39.1)	56 (37.3)	85 (40.3)	0.648
	≥400ug/mL	220 (60.9)	94 (62.7)	126 (59.7)	
Tumor diameter	<10 cm	207 (57.3)	98 (65.3)	109 (51.7)	0.013
	≥10 cm	154 (42.7)	52 (34.7)	102 (48.3)	
Preoperative TACE	TACE	103 (28.5)	40 (26.7)	63 (29.9)	0.587
	Non-TACE	258 (71.5)	110 (73.3)	148 (70.1)	
Edmondson Grade (%)	I+II	57 (15.8)	29 (19.3)	28 (13.3)	0.158
	III+IV	304 (84.2)	121 (80.7)	183 (86.7)	
MVI (%)	No	141 (39.1)	69 (46.0)	72 (34.1)	0.030
	Yes	220 (60.9)	81 (54.0)	139 (65.9)	
Tumor capsule (%)	Complete	84 (23.3)	39 (26.0)	45 (21.3)	0.363
	Absent or Partial	277 (76.7)	111 (74.0)	166 (78.7)	
Extent of liver resection	Major liver resection	232 (64.3)	94 (62.7)	138 (65.4)	0.672
	Minor liver resection	129 (35.7)	56 (37.3)	73 (34.6)	
Type of liver resection	Anatomical	139 (38.5)	59 (39.3)	80 (37.9)	0.870
	Non-anatomical	222 (61.5)	91 (60.7)	131 (62.1)	
Postoperative TACE	No	181 (50.1)	76 (50.7)	105 (49.8)	0.950
	Yes	180 (49.9)	74 (49.3)	106 (50.2)	
Site of recurrence	Intrahepatic	217 (78.9)	80 (76.9)	137 (80.1)	0.780
	Extrahepatic	28 (10.2)	11 (10.6)	17 (9.9)	
	Intrahepatic and extrahepatic	30 (10.9)	13 (12.5)	17 (9.9)	

TACE, transcatheter arterial chemoembolization; AST, aspartate aminotransferase; ALT, alanine aminotransferase; GGT, gamma glutamyl transpeptidase; ALP, alkaline phosphatase; Alb, albumin; TIBL, total bilirubin; DIBL, direct bilirubin; CR, creatinine; INR, international normalized ratio; PLT, blood platelet; AFP, alpha fetoprotein; HBV, hepatitis B virus; HCV, hepatitis C virus; MVI, microvascular invasion.

### Comparison of Perioperative Complications Between Patients With Preoperative TACE and Those Without Preoperative TACE

We compared the effects of preoperative TACE on perioperative complications and mortality. We found that preoperative TACE did not increase perioperative mortality, liver failure, bile leakage, ascites, wound infection, and other complications compared to patients without preoperative TACE (*P* > 0.05; **Table 8**).

**Table 8:** Comparison of perioperative complications between patients with preoperative TACE and those without preoperative TACE.

Variable		Overall (361)	Non-TACE (n=258)	TACE (n=103)	*p*
PLF (%)	No	344 (95.3)	243 (94.2)	101 (98.1)	0.196
	Yes	17 (4.7)	15 (5.8)	2 (1.9)	
Abdominal hemorrhage (%)	No	357 (98.9)	254 (98.4)	103 (100.0)	0.475
	Yes	4 (1.1)	4 (1.6)	0 (0.0)	
Bile leakage (%)	No	351 (97.2)	249 (96.5)	102 (99.0)	0.337
	Yes	10 (2.8)	9 (3.5)	1 (1.0)	
Incisional infection (%)	No	330 (91.4)	233 (90.3)	97 (94.2)	0.329
	Yes	31 (8.6)	25 (9.7)	6 (5.8)	
Organ/space infection (%)	No	339 (93.9)	242 (93.8)	97 (94.2)	1
	Yes	22 (6.1)	16 (6.2)	6 (5.8)	
Respiratory infection (%)	No	353 (97.8)	252 (97.7)	101 (98.1)	1
	Yes	8 (2.2)	6 (2.3)	2 (1.9)	
Pleural effusion (%)	No	316 (87.5)	230 (89.1)	86 (83.5)	0.196
	Yes	45 (12.5)	28 (10.9)	17 (16.5)	
Ascites (%)	No	327 (90.6)	236 (91.5)	91 (88.3)	0.473
	Yes	34 (9.4)	22 (8.5)	12 (11.7)	
Other complications (%)	No	351 (97.2)	252 (97.7)	99 (96.1)	0.646
	Yes	10 (2.8)	6 (2.3)	4 (3.9)	
	Absent or Partial	277 (76.7)	111 (74.0)	166 (78.7)	

TACE, transcatheter arterial chemoembolization; PLF, postoperative liver failure.

Comparison of clinicopathological characteristics and perioperative outcomes between patients with and without preoperative TACE in the total population.

## Discussion

TACE has been one of the most effective and safest local treatment for patients with unresectable HCC (8). Its use of embolic material to occlude the main blood vessels that will supply the tumor leads to ischaemic necrosis of the tumor and the chemotherapeutic drugs that can effectively kill the tumor tissue or tumor cells. In the last 20 years, many scholars have also applied TACE in the preoperative adjuvant treatment of large HCC (6–25, 33–36). The main objectives of preoperative TACE were: (1) induce tumor volume shrinkage and convert unresectable HCC into resectable HCC; (2) reduce postoperative tumor recurrence and improve long-term patient survival; (3) improve the detection of occult lesions not detected by preoperative imaging beyond TACE (6, 37). However, the effectiveness of preoperative TACE has been controversial (6–25, 33–36), and some scholars believe that preoperative TACE may only be benefit for certain specific groups of HCC, such as patients with large tumor diameters, multiple nodes and invasive HCC (8, 14, 26). However, there is no consensus on which type of HCC patients can benefit from TACE, so there is an urgent need to explore a reliable preoperative indicator to guide preoperative TACE.

CTCs are malignant tumor cells that invade into the peripheral blood *via* epithelial-mesenchymal (EMT) form, which reflect the tumor’s aggressiveness and is often used for prognostic monitoring in breast, colorectal, and prostate cancers (29, 30, 38, 39). CTCs testing is considered to be a reliable means of early screening for cancer, postoperative recurrence, or metastasis monitoring in HCC patients (40). There are various methods on the market to detect circulating tumor cells, among them the Cyttel method (30–32, 41, 42) and CellSearch™ are the most common (27, 28, 43). The CellSearch™ system assay uses the traditional EpCAM-dependent enrichment method to identify CTCs (41, 44), which has certain limitations. The most important point is that not all peripheral blood CTCs of HCC patients express EpCAM, only 30-40% of HCC cells express EpCAM (45). This results in the low sensitivity of the CellSearch™ system to detect CTCs (44). To overcome this problem, we used a negative immunomagnetic particle method to detect CTCs to improve the assay’s sensitivity. Our retrospective study found that patients with positive CTCs had shorter RFS and OS than those with negative CTCs, and the landmark analysis also found that CTCs status was associated with early postoperative recurrence (*P <* 0.05), possibly by causing early recurrence leading to patient death. These findings are consistent with recent studies (46–50).

In addition, we divided the overall population into CTC-positive group and negative-group based on CTCs status and explored whether patients in each group would benefit from preoperative TACE. This study suggested that preoperative TACE may prolong survival prognosis by reducing RFS in the CTC-positive population. At the same time, we also showed that non-preoperative TACE was a risk factor for RFS and OS in HCC patients by univariate and multivariate Cox regression analyses. However, in the CTC-negative group, preoperative TACE was not found to reduce postoperative recurrence and improve survival prognosis, and univariate and multivariate Cox regression analyses also showed that preoperative TACE did not improve long-term prognosis by reducing early recurrence in HCC patients but not affecting late recurrence.

Many studies have suggested that early postoperative recurrence of HCC may be associated with occult micrometastases remaining in the liver (26, 50, 51), and many factors influence the patient’s early postoperative tumor recurrence, including CTCs status, tumor diameter, tumor number, microvascular invasion, incomplete tumor envelope and satellite nodules (26, 50, 51). In this study, we found that patients with positive CTCs had a relatively larger tumor diameter (*P* < 0.05) and a higher positive rate of MVI (*P* < 0.05), so we hypothesized that the proportion of patients with occult metastases was higher in the CTC-positive group (52). As surgical resection alone does not remove residual occult foci, preoperative TACE can theoretically remove it. This also explains why preoperative TACE reduce early recurrence in CTC-positive patients and prolongs survival prognosis of patient (26, 28, 52). Second, consider that the vast majority of early recurrence are intrahepatic recurrence. According to the “seed” and “soil” theory of HCC recurrence and metastasis after surgery, preoperative TACE causes changes in the tumor microenvironment of hepatocellular carcinoma. Preoperative TACE may act as a herbicide, making it difficult for CTCs (seeds) to grow in the residual liver (soil) (52). Therefore, preoperative CTC testing is relevant to guide preoperative TACE treatment. In the comprehensive management of hepatocellular carcinoma, clinicians need to pay more attention to the clinical value of preoperative CTC testing. For CTC-positive patients, preoperative TACE is necessary to reduce early postoperative recurrence and prolong OS. However, for patients with CTC-negative, preoperative TACE may not be necessary.

In addition to analyzing the impact of preoperative TACE on the prognosis of HCC patients, we also evaluated the impact of perioperative complications of the subsequent surgery with preoperative TACE. The results found that preoperative TACE did not increase the complications such as liver failure, postoperative ascites, and associated postoperative infections (*P* **> 0.05**). Some papers reported that the effect of preoperative TACE on surgery was rare if the interval between preoperative TACE and surgery was more than four weeks (8). To be precise, the median time from preoperative TACE to surgical resection at our affiliated medical centre is 4.5 weeks (range 3-6 weeks). Secondly, liver resection is only performed by an experienced team of surgeons. The above results may minimise the impact of preoperative TACE in the perioperative period.

Our research has limitations. Firstly, this study is a single-center retrospective study with few cases. Therefore, in the follow-up study, we will conduct a multi-center, large sample prospective study with multiple medical centers to further demonstrate the value of CTC testing as a guide for preoperative TACE. Secondly, most of the population we include were infected with HBV, whereas most HCC patients in western countries are caused by factors such as HCV or alcohol. The result may not be suitable for Western populations.

In conclusion, this study suggests for the first to propose that preoperative CTC testing is a guide to predicting the efficacy of preoperative TACE for HCC. For patients with positive preoperative CTCs, preoperative TACE may be a reliable means to prevent early recurrence and improve patients’ postoperative prognosis.

## Data Availability Statement

The raw data supporting the conclusions of this article will be made available by the authors, without undue reservation.

## Ethics Statement

The studies involving human participants were reviewed and approved by Zhongshan Hospital Affiliated to Guangdong Medical University, Tongji Hospital of Tongji Medical College of Huazhong University of Science and Technology, Huangshi Central Hospital of Edong Healthcare Group, Hubei Polytechnic University, Xiaogan Central Hospital, General Hospital of Central Theater, Qinghai University Affiliated Hospital, and Renmin Hospital of Wuhan University. The patients/participants provided their written informed consent to participate in this study.

## Author Contributions

QZ wrote the paper. WH, QZ, and AM provided the data. FX analysed the data. XF, JC, and WZ reviewed and edited the manuscript. All authors read and approved the manuscript.

## Conflict of Interest

The authors declare that the research was conducted in the absence of any commercial or financial relationships that could be construed as a potential conflict of interest.

## Publisher’s Note

All claims expressed in this article are solely those of the authors and do not necessarily represent those of their affiliated organizations, or those of the publisher, the editors and the reviewers. Any product that may be evaluated in this article, or claim that may be made by its manufacturer, is not guaranteed or endorsed by the publisher.

## Supplementary Material

Supplementary Figure 1 Comparison of overall survival **(A)** and recurrence free survival **(B)** between CTC-positive and CTC-negative patients. Analysis of the effect of CTC status on early and late postoperative recurrence in overall HCC patients by landmark method.

Supplementary Figure 2 Analysis of the effect of CTC status on early and late postoperative recurrence in overall HCC patients by landmark method.
