# Supplementary figures and images for: Guiding Value of Circulating Tumor Cells for Preoperative Transcatheter Arterial Embolization in Solitary Large Hepatocellular Carcinoma: A Single-Center Retrospective Clinical Study
Source: Front Oncol. 2022 May 18;12:839597. doi: 10.3389/fonc.2022.839597 (PMC9159764; doi:10.3389/fonc.2022.839597)

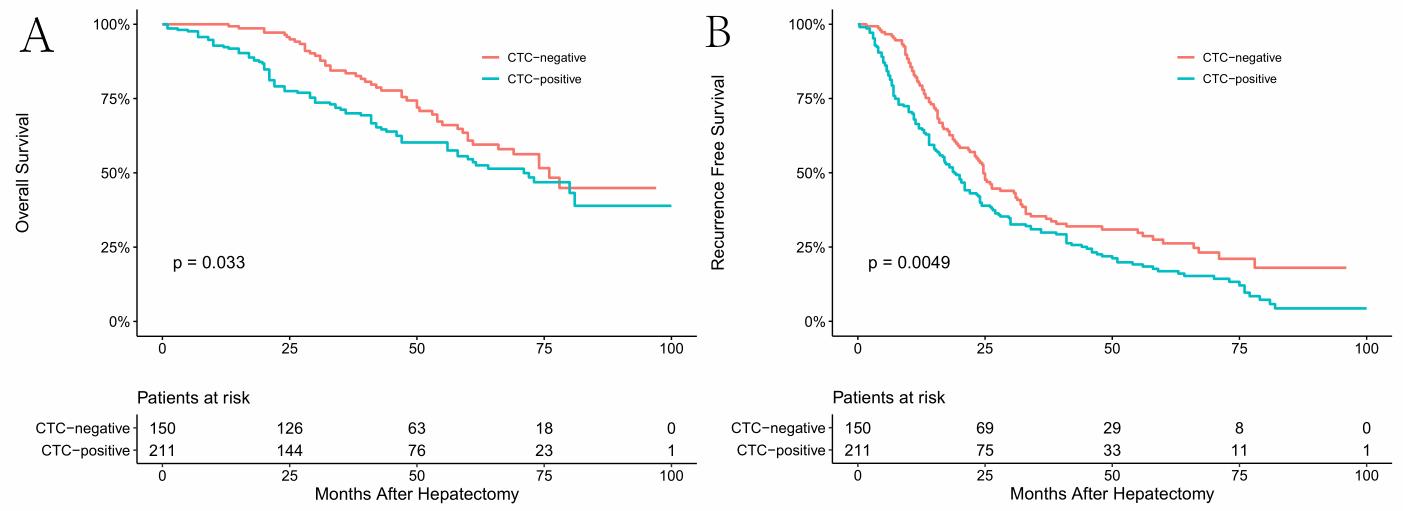

Supplement: Supplementary Figure 1 — Comparison of overall survival (A) and recurrence free survival (B) between CTC-positive and CTC-negative patients. Analysis of the effect of CTC status on early and late postoperative recurrence in overall HCC patients by landmark method. [file Image_1.jpeg]

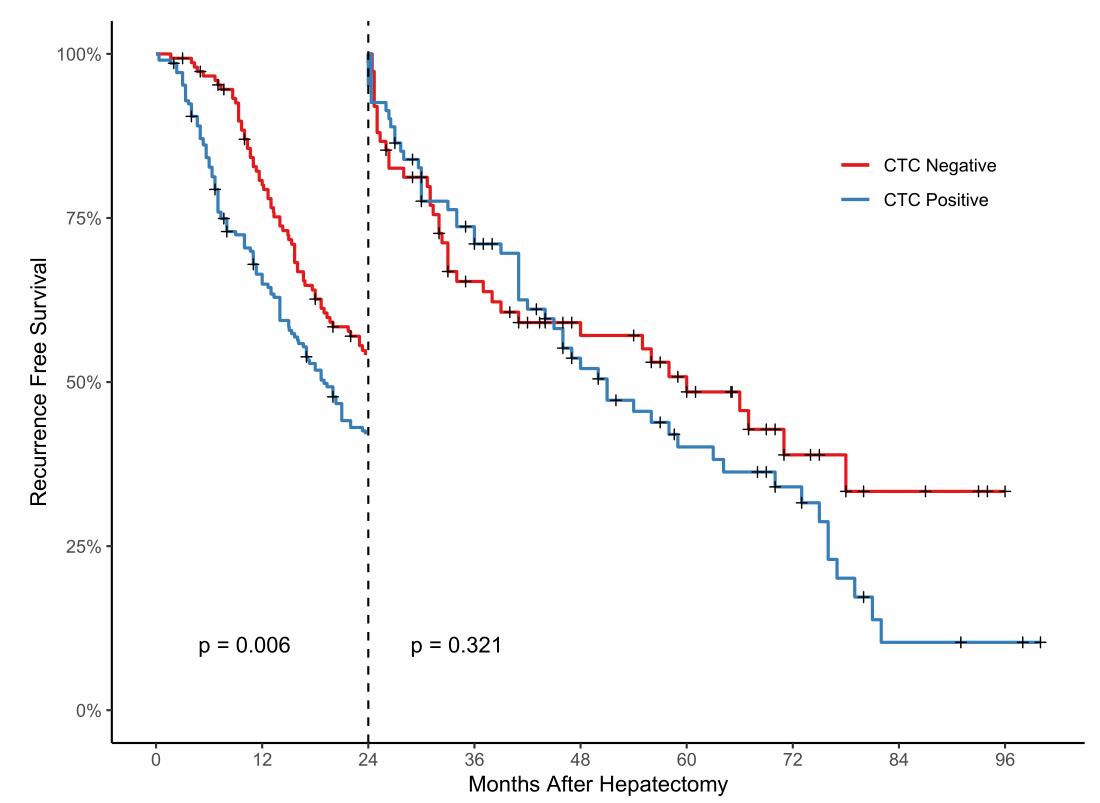

Supplement: Supplementary Figure 2 — Analysis of the effect of CTC status on early and late postoperative recurrence in overall HCC patients by landmark method. [file Image_2.jpeg]
